# Can the general public use vignettes to discriminate between Alzheimer’s disease health states?

**DOI:** 10.1186/s12877-016-0207-4

**Published:** 2016-02-03

**Authors:** Mark Oremus, Feng Xie, Eleanor Pullenayegum, Kathryn Gaebel

**Affiliations:** School of Public Health and Health Systems, University of Waterloo, 200 University Avenue West, Waterloo, ON Canada; Department of Clinical Epidemiology and Biostatistics, McMaster University, 1280 Main Street West, Hamilton, ON Canada; Program for Health Economics and Outcome Measures (PHENOM), 50 Charlton Avenue East, Hamilton, ON Canada; Centre for Evaluation of Medicines, St. Joseph’s Healthcare Hamilton, 25 Main Street West, Hamilton, ON Canada; Child Health Evaluative Sciences, Hospital for Sick Children, 555 University Avenue, Toronto, ON Canada

**Keywords:** Alzheimer’s disease, Clinical vignette, EQ-5D-5L, General public, Proxy, Quality-of-life – Alzheimer’s Disease

## Abstract

**Background:**

Valid estimates of health-related quality-of-life (HRQoL) are often difficult to obtain from persons with Alzheimer’s disease (AD) and family caregiver proxies. To help assess whether the general public can serve as an alternate source of proxy HRQoL estimates in AD, we examined whether the general public can use vignettes to discriminate between AD health states.

**Methods:**

We administered a telephone survey to randomly recruited participants from the general public who were aged 18 years or older. Interviewers read vignettes describing the mild, moderate, and severe AD health states to the participants, who answered the EQ-5D-5L and Quality of Life-Alzheimer’s Disease (QoL-AD) scales as if they had AD based on the vignette descriptions. Participants also answered the EQ-5D-5L for their current health states. We converted EQ-5D-5L responses into health utility scores using Canadian preference weights. We employed the Wilcoxon signed rank test to examine whether mean health utility scores and mean QoL-AD scores differed between health states. We used Pearson’s *r* to assess correlations between health utility and QoL-AD scores.

**Results:**

Forty-eight participants (median age = 53 years; 25 female) completed the telephone interview; health utility and QoL-AD scores decreased as AD severity increased (*p* <0.0001). Mean health utility scores were 0.65 (mild), 0.51 (moderate), and 0.25 (severe). Mean QoL-AD scores were 26.7 (mild), 23.0 (moderate), and 17.4 (severe). The correlations between health utility and QoL-AD scores were moderate to strong (*r* ≥ 0.62).

**Conclusions:**

Using the vignettes, the general public provided HRQoL estimates that discriminated between the three AD health states. This finding suggests the general public may be a promising source of proxy HRQoL estimates in place of persons with AD.

**Electronic supplementary material:**

The online version of this article (doi:10.1186/s12877-016-0207-4) contains supplementary material, which is available to authorized users.

## Background

The World Health Organization defines health-related quality-of-life (HRQoL) as an “individual’s perception of their position in life in the context of the culture and value systems in which they live and in relation to their goals, expectations, standards and concerns” [1]. In Alzheimer’s disease (AD), HRQoL is typically measured with validated scales such as the EuroQoL Group’s utility-based EQ-5D-5L scale [2] and the Quality-of-life – Alzheimer’s Disease (QoL-AD) scale [3]. Individuals’ responses to utility-based instruments such as the EQ-5D-5L are used to calculate health utility scores, while responses to the QoL-AD are used to calculate numerical scores that quantify HRQoL.

Health utility scores are used to compute quality-adjusted life-years (QALYs) [4] for economic evaluations of new health technologies. Economic evaluations are becoming an increasingly important component of public health insurance coverage decisions in AD and other disease areas. In the United Kingdom (UK), policy makers used an economic evaluation to recommend delisting coverage of cholinesterase inhibitors for persons with mild AD [5].

Due to cognitive impairment, many persons with AD may be unable to complete HRQoL scales, especially as the disease progresses and cognitive function worsens [6, 7]. Although some persons with AD might be capable of answering the question “How do you feel now?” [8], the verbal or non-verbal responses to this question cannot be converted into health utility scores or QALYs.

Researchers frequently ask family caregivers to provide proxy HRQoL estimates in place of their loved ones with AD. However, family caregivers may underestimate their loved ones’ HRQoL because they integrate their own life experiences (e.g., the burden and stress of caregiving) into the proxy assessments [9–15].

Given the challenges of obtaining valid HRQoL estimates from persons with AD or their caregiver proxies, we launched a research program to investigate whether the general public can serve as an alternate source of valid proxy HRQoL estimates in AD. The first step in this research program was to examine whether the general public could provide HRQoL estimates that discriminate between mild, moderate, and severe AD. This paper reports the findings of this first step.

## Methods

### Vignettes

Most members of the general public are unlikely to have first-hand knowledge of AD. To provide a frame of reference, we developed three vignettes that describe what life is like for an individual who lives with mild, moderate, or severe AD (Additional file 1). We formulated the vignettes through content analysis [16] of feedback from focus groups composed of persons with AD, family caregivers, and physicians who specialize in treating AD. Members of the general public then read each vignette and answered the EQ-5D-5L and QoL-AD as if they had AD based on the vignette descriptions.

### Participant recruitment and interview administration

We used published telephone numbers and random digit dialling to recruit a general public sample from the Greater Toronto Area, Niagara Region, and City of Hamilton (Ontario, Canada). We included individuals aged 18 years or older who could communicate in English. Participants were randomly allocated to hear the mild, moderate, and severe vignettes in one of the six possible orders of vignettes during a telephone interview.

The telephone interviews followed a fixed script administered using computer-assisted telephone interview software (Telephone Survey Software – Voxco Canada, Montréal, QC). In the first stage of the interview, the interviewers asked participants for their age, sex, highest education level, and annual household income. Participants also indicated whether any of their family members or close friends had been diagnosed with AD. Participants rated their own HRQoL on the interview day by completing the EQ-5D-5L. In the second stage of the interview, the interviewers read the vignettes to participants. After hearing each vignette, participants completed the EQ-5D-5L and the Qol-AD as if they had AD according the description just heard.

### Health-related Quality-of-life Scales

The EQ-5D-5L consists of five questions about mobility, self-care, usual activities, pain/discomfort, and anxiety/depression [2]. Each question has five response levels: no impairment (level 1), slight impairment (level 2), moderate impairment (level 3), severe impairment (level 4), and extreme impairment (level 5). We used a Canadian-based algorithm [17] to transform each participant’s EQ-5D-5L responses into a single health utility score between 0 and 1. A score of 0 represents a health state equivalent to being dead and a score of 1 represents perfect health [4].

The QoL-AD measures the quality-of-life of persons with AD [3]. Quality-of-life is rated on each of 13 domains and response options range from ‘poor’ (score = 1) to ‘excellent’ (score = 4). The overall scale score ranges from 13 to 52 (higher scores indicate better quality-of-life). Higher QoL-AD scores are correlated with less behavioural impairment, better psychological status, less impairment on physical function, and better interpersonal environments [3].

### Statistical analysis

We employed the Wilcoxon signed rank test to examine whether the differences in health utility scores between vignettes were statistically significant. We used the same test to investigate whether the differences in QoL-AD scores between vignettes were statistically significant. We employed Pearson’s *r* to assess the correlation between each vignette’s health utility and QoL-AD scores. The analyses were implemented in SPSS v22 (IBM Corporation, Armonk, NY).

For sample size, available resources allowed us to plan on recruiting 48 participants. To determine the smallest differences in score between vignettes that we could detect with this number, we undertook a sample size calculation based on the paired t-test, setting α = 0.05 and 1-ß (power) = 0.84 (equivalent to 80 % power after adjusting for the lower efficiency of the Wilcoxon signed rank test compared to the paired t-test [18]). We used data from previous research [19] to compute standard deviations for mean differences in health utility and QoL-AD scores across AD health states (i.e., 0.04 [health utility], 1.47 [QoL-AD]). The calculations showed that 48 participants would allow us to detect differences in health utility score of at least 0.02 and differences in QoL-AD score of at least 0.64. Thus, we would be able to detect differences in health utility score that were smaller than the minimum important difference of 0.074 [20] (the literature does not report a minimum important difference for the QoL-AD). For the correlation analysis, 48 participants would permit us to detect at least a ‘fair’ correlation (*r* ≥ 0.4) between health utility and QoL-AD scores at α = 0.05 and 1-ß = 0.8.

Prior to commencing the study, we obtained approval from the Hamilton Integrated Research Ethics Board (reference number 13-271). All participants gave oral consent to participate in this study at the start of the interviews.

## Results

### Sample characteristics

The median age of the 48 participants was 53 years and 25 were female (Table 1). The majority of participants (*n* = 34) were college or university graduates and most (*n* = 28) reported annual household incomes of at least $60,000. Twenty-three participants said they knew a family member or close friend with AD. The mean health utility score for the participants’ current health state was 0.87 (95 % confidence interval [CI]: 0.83 to 0.91).

**Table 1:** Sample characteristics

Characteristic	Data
Age – median (25^th^–75^th^ percentiles)	53.0 y (34.5 y–67.8 y)
Sex – *n*	
Female	25
Male	23
Education – *n*	
Less than high school^a^	2
High school graduate^a^	8
Technical or trade school graduate^a^	4
College graduate	21
University graduate (Bachelor, Master’s, Doctorate)	13
Annual household income^b^ – *n*	
<$20,000^a^	2
$20,000–$39,999^a^	6
$40,000–$59,999^c^	6
$60,000–$79,999^c^	6
≥ $80,000	22
Missing	6
Family member/close friend diagnosed with AD – *n*	
Yes	23
No	25
Health utility index score – mean (95 % CI)	0.87 (0.83–0.91)

*AD* Alzheimer’s disease, *CI* confidence interval, *n* number of participants, *y* years

^a^Categories combined with one another for regression analysis

^b^Canadian dollars

^c^Categories combined with one another for regression analysis

Participants’ responses to the EQ-5D-5L shifted from less to more problems as health states declined (Table 2). For participants’ current health state, most responses were concentrated in the ‘no problem’ response category. For mild AD, most responses indicated ‘no’ or ‘slight’ problems; for moderate AD, the bulk of responses reflected ‘slight’ or ‘moderate’ problems. For severe AD, the majority of participants answered ‘severe’ or ‘extreme’ problems. These trends were less pronounced for the ‘pain/discomfort’ question relative to the other four questions, probably because the vignettes did not describe pain-related symptoms.

**Table 2:** EQ-5D-5L Responses

	Health state			
	Current^a^	Mild AD	Moderate AD	Severe AD
Mobility – *n*				
No problems	38	13	8	0
Slight problems	6	21	13	6
Moderate problems	3	13	21	17
Severe problems	1	0	4	17
Unable to walk	0	1	2	8
Self-care – *n*				
No problems	45	2	3	1
Slight problems	2	24	8	0
Moderate problems	1	16	27	11
Severe problems	0	5	8	18
Unable to wash/dress	0	1	2	18
Usual Activities – *n*				
No problems	41	3	0	0
Slight problems	4	12	5	2
Moderate problems	2	27	26	8
Severe problems	0	5	14	23
Unable to do	1	1	3	15
Pain/Discomfort – *n*				
No pain/discomfort	22	19	15	7
Slight pain/discomfort	21	20	13	13
Moderate pain/discomfort	4	9	18	16
Severe pain/discomfort	1	0	1	11
Extreme pain/discomfort	0	0	1	1
Anxiety/Depression – *n*				
Not anxious/depressed	32	4	2	1
Slightly anxious/depressed	13	14	6	6
Moderately anxious/depressed	2	18	19	11
Severely anxious/depressed	1	3	17	15
Extremely anxious/depressed	0	1	4	14
Refused to answer	0	1	0	1

*AD* Alzheimer’s disease, *n* number of participants

^a^Participants’ current health state on the day of the interview

Given five questions and five response categories, the EQ-5D-5L contains 3125 possible response combinations. In our study, the participants reported 15 distinct combinations for their current health state, 38 for mild AD, 43 for moderate AD, and 42 for severe AD. Sixteen participants reported that their current health state entailed no problems on all five questions, while a further 15 participants reported no problems on four questions and slight problems on one question. None of the participants reported no problems on all five questions for any of the AD health states.

### Health-related quality-of-life estimates for Alzheimer’s disease

Participants’ HRQoL estimates were inversely related to disease severity (Table 3). Mean health utility scores were 0.65 (mild), 0.51 (moderate), and 0.25 (severe). Mean QoL-AD scores were 26.7 (mild), 23.0 (moderate), and 17.4 (severe). All of the scores were statistically significantly different from one another (*p* <0.0001). On average, participants’ health utility scores for their own current health state were higher than their utility scores for mild AD (mean difference: 0.23; *p* <0.0001).

**Table 3:** Health-related quality-of-life estimates for Alzheimer’s disease

Health state	EQ-5D-5L^a^	QoL-AD^b^
	Mean	Mean
Mild	0.65	26.7
Moderate	0.51	23.0
Severe	0.25	17.4
Difference: Mild - Moderate	0.15^c^	3.7^c^
Difference: Mild - Severe	0.41^c^	9.3^c^
Difference: Moderate - Severe	0.27^c^	5.6^c^

*CI* confidence interval, *QoL-AD* Quality-of-life – Alzheimer’s Disease

^a^EQ-5D-5L responses were converted into health utility scores (0 = death; 1 = perfect health); means and mean differences were rounded to two decimal places

^b^Score range 13 to 52: lower scores indicate poorer health-related quality-of-life

^c^ *p* <0.0001

**Fig. 1:**
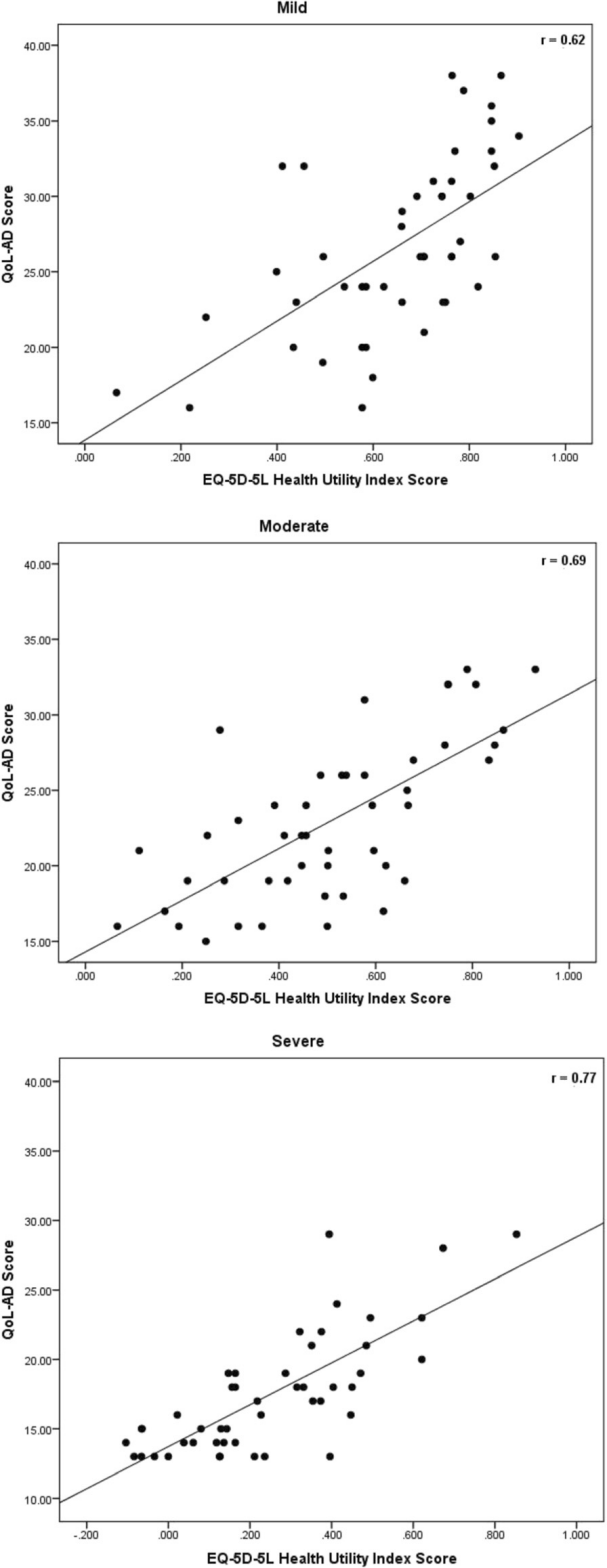
Correlations between general public health utility scores and QoL-AD scores. r: Pearson correlation coefficient; QoL-AD: Quality-of-life – Alzheimer’s Disease scale

The correlations between health utility and QoL-AD scores were moderate to strong (Fig. 1): *r* = 0.62 (*p* <0.0001) for the mild AD vignette, *r* = 0.69 (*p* <0.0001) for the moderate vignette, and *r* = 0.77 (*p* <0.0001) for the severe vignette.

## Discussion

The study participants, all members of the general public, provided HRQoL estimates that discriminated between the mild, moderate, and severe AD health states. Participants reported more problems on the EQ-5D-5L as they responded to vignettes describing successively worsening AD health states. Also, most participants provided unique EQ-5D-5L response combinations for each AD health state, which suggests that the vignettes were not leading individuals into a narrow band of responses. Health utility scores and QoL-AD scores declined as health states worsened. Correlations between the EQ-5D-5L and QoL-AD were moderately strong, which shows that participants’ responses were robust across different measurement instruments.

Only two other published AD studies have examined the topic that we present in this paper. In our earlier research, we conducted a vignette-based study and elicited HRQoL estimates from a single-city convenience sample of 100 members of the general public [19]. Although we found an inverse relation between participants’ HRQoL estimates (EQ-5D-5L and QoL-AD) and the severity of the AD, each participant provided HRQoL estimates for a single health state, rather than for all three health states. Also, we wrote the vignettes featured in the earlier study without input from other stakeholders because the intent of this project was to conduct an initial exploration of whether the general public can understand and appreciate the intricacies of a syndrome such as AD. Our current work replicates these earlier findings using a different set of vignettes, which demonstrates that the general public’s HRQoL estimates are not tied to the specific vignettes used in a study. Furthermore, since the participants in the current study provided HRQoL estimates for all three health states, random allocation to the sequence in which the health states were presented was shown to control for ordering effects.

In the second published AD study [21], 78 members of the general public in the UK used the time trade-off task [22] to value eight of 1024 possible health states from the DEMQOL-U and eight of 256 possible health states from the DEMQOL-U-Proxy. The DEMQOL instruments [23] measure HRQoL in dementia, with the DEMQOL-U intended to be completed by persons with AD and the DEMQOL-U-Proxy intended to be completed by caregivers. While participants’ health utility scores tended to decrease as DEMQOL health states worsened, the researchers did not tell participants that they were rating health states for dementia. Indeed, three of the domains on the DEMQOL-U (i.e., cheerfulness, frustration, loneliness) and three of the domains on the DEMQOL-U-Proxy (i.e., liveliness, keeping oneself looking nice, frustration) have no obvious links to dementia. Therefore, the UK participants’ HRQoL estimates may have reflected their own health states on the day of the study interview, rather than any health states related to dementia. In our study, the interviewers told participants that they were estimating HRQoL for AD health states, which is comensurate with methods guidance [24].

Researchers in other illness domains have found that members of the general public can discriminate between different health states (i.e., hepatic encephalopathy [25]. non-small cell lung cancer [26], rheumatoid arthritis [27]) after being presented with vignettes to describe the health states. In another study [28], the researchers used data from randomized controlled trials to develop ‘before-after’ vignettes for each of five health conditions (i.e., depression, osteoarthritis, insomnia, chronic obstructive pulmonary disorder, Crohn’s disease). The ‘before’ vignettes described the symptoms for each condition prior to treatment; the ‘after’ vignettes described a less severe set of symptoms for each condition following treatment. Post-treatment utilities were statistically significantly lower than pre-treatment utilities for all of the health conditions except insomnia.

### Implications of the research

Our current and past work [19] has consistently shown that members of the general public can discriminate between the mild, moderate, and severe AD health states. The next step is to establish whether the general public can provide valid proxy HRQoL estimates in place of persons with AD. To investigate this issue, we plan to compare the general public’s proxy HRQoL estimates to estimates obtained directly from persons with AD. A challenge will be to recruit persons with AD who are cognitively capable of answering the EQ-5D-5L or QoL-AD.

The use of the general public instead of caregivers to measure HRQoL can have practical advantages for researchers. General public samples are far more accessible than caregiver samples. Members of the general public can be recruited through numerous vehicles, including ongoing population-level studies (e.g., Canadian Longitudinal Study on Aging [CLSA] [29]) or telephone polling surveys.

AD caregivers would be a challenge to recruit through either vehicle. Population studies may not ask participants about their caregiver status, nor may they be designed to recruit caregivers. Surveys conducted through polling firms would require screening questions to identify caregivers from among the people telephoned and invited to participate. The number of telephone calls required to recruit a sufficient number of caregivers could be substantial enough to exceed the resource capacity of many researchers. Alternatively, researchers could attempt to build rosters of caregivers in collaboration with medical practices, advocacy organizations, support groups, or long-term care facilities. However, this approach takes time and is compounded by the fact that recruitment partners do not always prioritize research activities.

If we find the general public can provide valid proxy HRQoL estimates in AD, then these estimates can be employed in economic evaluations to calculate QALYs and help policy makers decide if public health insurers should reimburse new AD treatments. Due to the relative ease of randomly recruiting general public samples and adminstering vignettes along with the EQ-5D-5L and QoL-AD, researchers will be able to collect data from samples based in the jurisdictions to which policy decisions will be applied. The greater difficulty of recruiting caregivers into research studies could otherwise lead analysts to use HRQoL data collected from other jurisdictions, perhaps simply as a matter of expediency given the time-sensitive nature of reimbursement decision making. Such data may not be readily transferable across jurisdictions because of socio-demographic differences between populations or differences in healthcare systems.

### Limitations

The study sample was over-representative of persons with a post-secondary education and half of the participants were over 53 years of age. More educated individuals might possess a greater capacity to distinguish between the nuances of the vignettes. Persons in middle or old age might be more aware of AD because age is one of the strongest risk factors for the disease. Less educated and younger samples may have a more difficult time of discriminating between AD health states.

Vignettes represent abstractions of real-life phenomena and do not capture the entire essence of living with disease. To enhance the validity of the vignettes employed in this study, we used input from stakeholders who experience AD on a daily basis to craft the content of the vignettes. We also point out that vignettes are used extensively in AD research to examine diverse samples’ attitudes to a multiplicity of issues such as therapeutic interventions and HRQoL [30]. Examples of diverse samples used in other vignette-based studies include persons with AD, caregivers, healthcare professionals, and members of the general public. Vignettes have also been used to examine people’s emotional reactions to AD, as well as ethno-cultural and social differences in their attitudes to the disease [30].

## Conclusions

Members of the general public can discriminate between AD health states. This finding suggests the general public might be a source of valid proxy HRQoL estimates in place of persons with AD. Further assessment of the general public’s potential to serve as a proxy will require comparing the general public’s HRQoL estimates to estimates obtained directly from persons with AD.

## Additional file

Additional file 1:
**Vignettes.** (DOCX 149 kb)

## Abbreviations

AD: Alzheimer’s disease
CI: confidence interval
CLSA: Canadian Longitudinal Study on Aging
HRQoL: health-related quality-of-life
QALY: quality-adjusted life-year
QoL-AD: Quality of Life-Alzheimer’s Disease scale
UK: United Kingdom
