# Supplementary material for: Can the general public use vignettes to discriminate between Alzheimer’s disease health states?
Source: BMC Geriatr. 2016 Feb 3;16:36. doi: 10.1186/s12877-016-0207-4 (PMC4738787; doi:10.1186/s12877-016-0207-4)
Supplement: Additional file 1: — Vignettes. (DOCX 149 kb) [file 12877_2016_207_MOESM1_ESM.docx]

**Mild Alzheimer’s disease**

If you have Alzheimer’s disease, then you might forget recent events of importance to you. For example, if you are interested in hockey, then you might forget whether your favorite team won their last game, although you may remember watching the game itself. You might forget the names of people you met recently, or the names of local streets. You may no longer be able to handle your personal finances or plan leisure activities such as dinner parties or vacations. Simple decisions such as what to order in restaurants might become difficult to make. Your ability to concentrate may decrease and you might have difficulty driving. You may start having trouble finding words to express your thoughts. You might need to be reminded to shower or brush your teeth, but you could do these activities on your own. You may need to post notes around the home to remind you of simple things like turning off the stove. You may remain aware of the day and your surroundings. You might be able to travel to familiar locations like the supermarket, although probably with the help of a loved one or friend. If you are in a social gathering, then you might have difficulty following the conversation. You may continue to enjoy hobbies such as puzzles or reading.

**Moderate Alzheimer’s disease**

If you have Alzheimer's disease, then you might forget or mix up the names of your grandchildren or in-laws, but you may remember the names of your spouse and children. Sometimes you may not know the date or day of the week. You may also forget where you are now or what you did recently. You might misplace common items like your glasses or the toothpaste. You may lose interest in doing many of the things that you once liked to do. You are unlikely to drive and may have difficulty using public transit on your own. If you do go out, then someone might have to guide you the entire time. You may not follow the flow of conversations in social gatherings. You would need help with things like choosing what clothing to wear, using the telephone or computer, paying bills, cleaning house, buying groceries, preparing meals, or taking medications. You might be able to get dressed, eat, and use the toilet on your own. You may experience behavior and mood changes, such as becoming outgoing and talkative if you were once shy, or vice versa. You are probably incapable of making simple decisions like what to do on the weekend. You may still enjoy activities like listening to music or watching television. You may remember things that happened long ago, such as buying a doll with your mother or waking up at dawn to go to hockey practice with your father.

**Severe Alzheimer’s disease**

If you have Alzheimer’s disease, then you may be unable to do one or more of several basic chores on your own, including eating, dressing, bathing, toileting, looking after personal hygiene, or walking. You would probably need full-time help with what you cannot do and you would be unable to live alone. You may be incontinent. You may experience agitated behaviour, such as struggling with the person who is trying to bathe or dress you, even if this person is your spouse or child. You may forget one or more important details of your life, including your birthday, wedding anniversary, spouse’s name, or your children’s names. You may know who you are and what your name is; you might recognize your spouse and children without knowing exactly who they are. You may not know the day, date, or season of the year, and you may be unaware of where you are. You might be unable to go outside on your own and you may be too sick to be taken anywhere. Your ability to speak, as well as to hold and understand conversations, might be extremely limited. You are probably incapable of doing any chores or hobbies. You would probably not be able to make simple decisions like where to sit or what to do with your day. You may enjoy certain foods or passive activities like listening to music.
